# Fine-scale individualized gyral folding-based cortical similarity networks reveal distinct organizational patterns in Alzheimer’s disease and Lewy body dementia

**DOI:** 10.1162/IMAG.a.1322

**Published:** 2026-08-06

**Authors:** Minheng Chen, Chao Cao, Tong Chen, Dunia Alhamad, Tianming Liu, Li Su, Dajiang Zhu

**Affiliations:** Department of Computer Science and Engineering, University of Texas at Arlington, Arlington, TX, United States; Sheffield Institute for Translational Neuroscience, University of Sheffield, Sheffield, United Kingdom; King Saud Bin Abdulaziz University for Health Sciences (KSAU-HS), Ministry of National Guard Health Affairs, Riyadh, Kingdom of Saudi Arabia; School of Computing, University of Georgia, Athens, GA, United States; Department of Psychiatry, University of Cambridge, Cambridge, United Kingdom

**Keywords:** Alzheimer’s disease, Lewy body dementia, morphometric similarity networks, cortical folding, individualized brain networks

## Abstract

Alzheimer’s disease (AD) and Lewy body dementia (LBD) are common neurodegenerative dementias with overlapping clinical presentations, making differential diagnosis challenging. While structural magnetic resonance imaging (MRI) has revealed characteristic regional atrophy patterns, regional morphometric measures alone may not fully capture distributed cortical alterations. Morphometric similarity networks (MSNs) offer a systems-level framework to characterize coordinated structural organization, but existing approaches typically rely on atlas-based parcellations that may obscure individual-specific cortical folding geometry. Here, we propose a fine-scale, folding-informed cortical similarity network framework based on automatically detected three-hinge gyral (3HG) landmarks. Using a thickness-constrained arealization strategy in native surface space, we define individualized cortical regions and construct subject-specific MSNs without cross-subject registration. We then investigate how network topology relates to landmark-defined node count and how these properties differ between AD and LBD. We find that several graph theoretical metrics, particularly global efficiency and characteristic path length, exhibit clear associations with the number of detected landmarks, indicating that topology in individualized networks is partly shaped by node availability. When accounting for landmark count, several apparent group differences in global topology are attenuated, whereas multiple heterogeneity-related metrics remain significant, indicating that node-count scaling substantially influences the interpretation of individualized network topology. Nevertheless, multivariate topological patterns remain informative for AD/LBD classification after residualizing for node count, and landmark count itself provides modest diagnostic information. These findings highlight node-count scaling as a key methodological consideration in individualized structural networks and suggest that folding-based MSNs capture disease-related variation in cortical network organization between AD and LBD.

## 1 Introduction

Alzheimer’s disease (AD) and Lewy body dementia (LBD) are among the most common neurodegenerative dementias in older adults (McKeith et al., 2017; Walker et al., 2015). Despite distinct underlying pathologies, the two disorders often present with overlapping cognitive and clinical features, particularly in early disease stages. Symptoms such as memory decline, executive dysfunction, and cognitive fluctuations can occur in both conditions, making differential diagnosis challenging (E. J. Burton et al., 2004; Gnanalingham et al., 1997; McKeith et al., 2004, 2020). Accurate differentiation between AD and LBD is, therefore, crucial, as the two disorders differ in prognosis, treatment response, and disease mechanisms (Vemuri et al., 2011).

Structural magnetic resonance imaging (MRI) has been widely used to investigate disease-related brain changes in dementia. Traditional morphometric analyses typically focus on regional structural measures, including cortical thickness, cortical gray-matter volume, and subcortical volumetric measures. These approaches have revealed characteristic medial temporal atrophy in AD and relatively preserved hippocampal structures in LBD (E. Burton et al., 2009; Whitwell et al., 2008). However, regional morphometric measures alone may not fully capture distributed structural alterations across the cortex, particularly given the substantial inter-individual variability in cortical morphology (Borrell, 2018; Lin et al., 2021; Seidlitz et al., 2018). Increasingly, connectomics approaches have been proposed to bridge regional structural alterations and large-scale network organization, providing a systems-level perspective on neurodegeneration (Bullmore & Sporns, 2009; Fornito et al., 2016). Morphometric similarity networks represent one such framework, quantifying the similarity of structural features across cortical regions to characterize coordinated anatomical organization (Sebenius et al., 2025; J. Wang & He, 2024). These networks have been shown to reflect underlying cytoarchitectonic and connectivity patterns and can reveal disease-related alterations in cortical organization (Tijms et al., 2013; Yao et al., 2024; Yi et al., 2022). Despite their promise, most existing morphometric similarity studies rely on atlas-based cortical parcellations and cross-subject registration (W. Li et al., 2017; Liang et al., 2025; Sebenius et al., 2023). Such approaches may obscure individual-specific folding geometry and introduce potential alignment biases when comparing cortical structures across individuals (Fischl et al., 2008). Furthermore, atlas-based regions impose a fixed node definition that may not optimally capture fine-scale structural variation. Importantly, morphometric properties of the cortex are themselves strongly shaped by cortical folding geometry. Structural features such as cortical thickness vary systematically along the gyral–sulcal spectrum and are closely related to local curvature and cortical shape (Demirci & Holland, 2022). Cortical folding patterns reflect fundamental neurodevelopmental and biomechanical constraints shaping cortical architecture and are closely linked to underlying connectivity patterns and functional specialization (Fischl et al., 2008; Rakic, 1988; Van Essen, 1997). Consequently, cortical folding patterns may provide a natural scaffold for constructing structural similarity networks. Leveraging folding landmarks, therefore, offers an individualized and biologically meaningful basis for defining network nodes and modeling cortical similarity.

Recent studies, including our previous work (M. Chen, Chen, Cao, Zhang, Liu, et al., 2026; T. Chen, Chen, Zhang, et al., 2026; T. Chen, Chen, Zhuang, et al., 2025), have begun to explore the potential of folding-informed individualized networks for dementia analysis. Our previous work (M. Chen, Chen, Cao, Zhang, Liu, et al., 2026; T. Chen, Chen, Zhang, et al., 2026) has demonstrated that individualized structural networks derived from cortical folding landmarks can achieve promising performance in dementia classification using deep learning models. In particular, both cortical similarity networks constructed from morphometric features and fiber connectivity networks derived from diffusion MRI have been used to distinguish AD, LBD, and cognitively normal (CN) individuals using data-driven models. These studies suggest that folding-informed individualized networks capture diagnostically relevant structural patterns. However, predictive modeling alone provides limited insight into the biological mechanisms underlying these classification results. These approaches primarily focus on predictive performance and provide limited insight into the structural mechanisms underlying the observed classification results. In particular, it remains unclear which aspects of network organization contribute to disease differentiation and how network topology relates to the underlying cortical morphology. Understanding these mechanisms is important not only for interpreting predictive models but also for revealing biologically meaningful alterations in cortical organization associated with neurodegenerative diseases.

To move beyond purely predictive modeling, it is, therefore, important to characterize the topological organization of these individualized networks and to identify which aspects of network structure contribute to disease differentiation. Graph theoretical analysis provides a powerful framework for characterizing large-scale network organization (Rubinov & Sporns, 2010). Metrics such as clustering, characteristic path length, assortativity, and global efficiency capture complementary aspects of network topology and have been widely applied to investigate network alterations in neurodegenerative diseases (Stam et al., 2009; Tijms et al., 2013). However, an important methodological issue in network analysis is that graph metrics depend strongly on network size and density (Fornito et al., 2010; Van Wijk et al., 2010; Zalesky et al., 2010). When networks are constructed from individualized landmarks rather than fixed atlas nodes, the number of nodes may vary substantially across individuals, introducing scaling effects that can influence graph theoretical measures and complicate the interpretation of group differences. These issues are particularly relevant for networks derived from cortical folding landmarks, where landmark availability may vary due to both anatomical variability and disease-related structural changes, yet the impact of such node-count variability on network topology remains largely unexplored.

In this study, we develop a fine-scale gyral folding-based cortical similarity network framework to investigate structural network organization in Alzheimer’s disease and Lewy body dementia. Our approach constructs networks from automatically detected three-hinge gyral (3HG) landmarks, which represent key geometric points within cortical folding patterns. Using a thickness-constrained arealization strategy, local cortical regions surrounding each landmark are defined in the native cortical surface space. Morphometric similarity between these regions is then used to construct subject-specific cortical similarity networks. Notably, this framework enables individualized network construction without requiring atlas-based parcellation or cross-subject registration, thereby preserving subject-specific folding geometry. Using this framework, we investigate how network topology relates to the number of detected landmarks and how these properties differ between AD and LBD. Our analysis reveals three main findings. First, several graph theoretical metrics exhibit clear scaling relationships with the number of detected 3HG landmarks, indicating that network topology is closely linked to landmark availability in individualized folding-based networks. Second, when accounting for landmark count, some single-metric group differences are attenuated, suggesting that part of the observed topological variation reflects node-count effects. Third, multivariate topological patterns remained weakly but statistically significantly informative for AD/LBD classification after residualizing for node count (AUC ≈ 0.63; permutation p = 0.003), and node count itself provided modest diagnostic information. Together, these findings highlight node-count scaling as an important consideration in individualized structural network analysis and suggest that folding-based cortical similarity networks capture disease-related variation in cortical network organization.

## 2 Methods

**Fig. 1.**
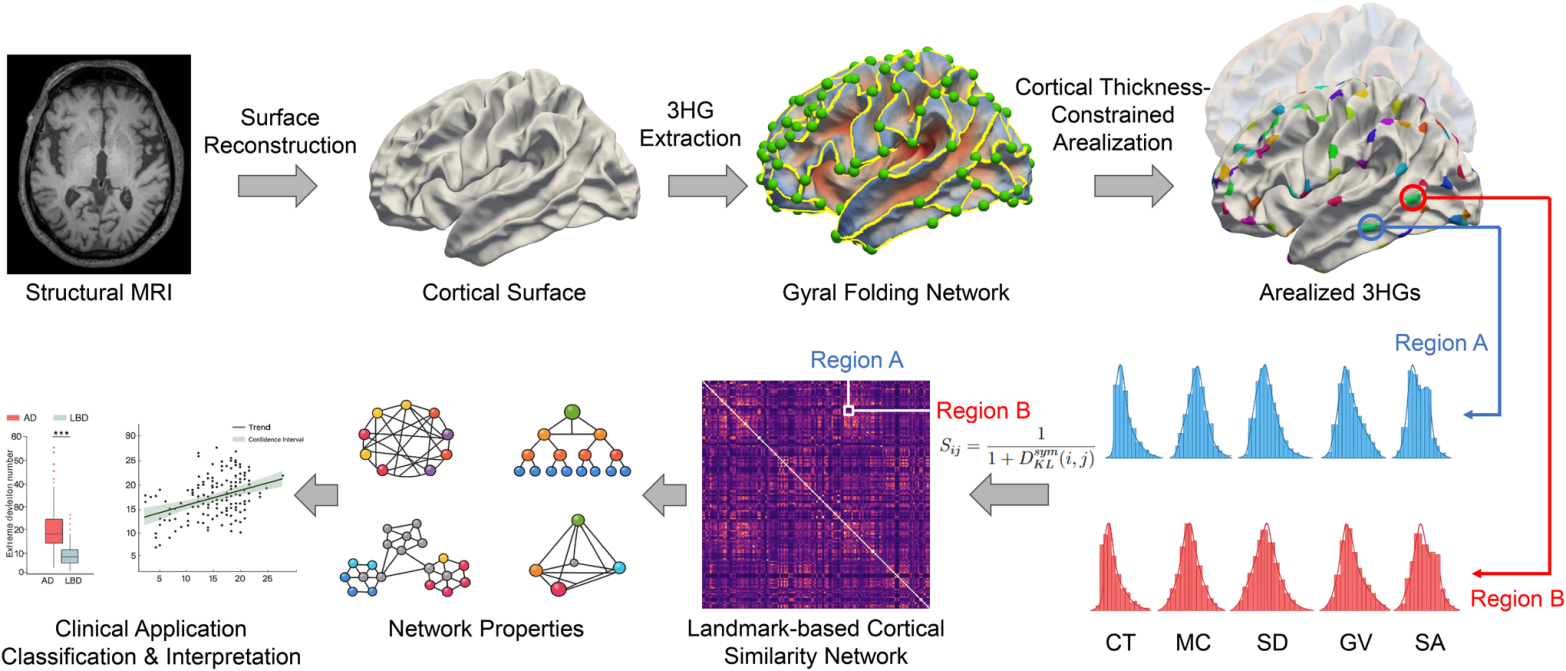
Overview of the proposed Gyral folding-based cortical similarity network framework. Structural MRIs are reconstructed into cortical surfaces, from which 3HGs are extracted as folding landmarks. Cortical thickness-constrained arealization defines localized regions around each landmark, and vertex-wise morphometric features are used to characterize regional feature distributions. Pairwise morphometric similarity between regions is computed using a symmetric KL divergence, producing an individualized landmark-based cortical similarity network for downstream clinical applications, including machine-learning-based classification and graph theoretical interpretation of disease-related cortical organization.

This study proposes a landmark-based cortical similarity network framework to characterize cortical structural organization (Fig. 1). Structural MR images were processed to reconstruct cortical surfaces and extract 3HGs as folding landmarks. Cortical thickness-constrained arealization defined localized regions around each landmark, from which multivariate morphometric features were used to construct individualized cortical similarity networks. The following sections describe the datasets, preprocessing, 3HG extraction, thickness-constrained arealization, cortical similarity network construction, and network metrics used for graph analysis.

### 2.1 Dataset

This study included participants diagnosed with AD and LBD, recruited from several multiple centers including community-dwelling population of patients referred to local Old Age Psychiatry, Geriatric Medicine, or Neurology services. Only subjects with available T1-weighted structural MRI scans were included, and demographic information was collected when available. Three cohorts of participants are (1) the Multimodal Imaging in Lewy Body Disorders (MILOS) project at the School of Clinical Medicine, University of Cambridge, in collaboration with Cambridge University Hospitals NHS Foundation Trust (CUH). The project is supported by Lewy Body Society, Addenbrooke’s Charitable Trust, and Alzheimer’s Research UK. Participants were recruited from three UK sites: Sheffield, Cambridge, and Nottingham. (2) A study funded by the Sir Jules Thorn Charitable Trust and the National Institute for Health Research (NIHR) Newcastle Biomedical Research Centre and the Biomedical Research Unit in Lewy Body Dementia based at Newcastle upon Tyne Hospitals National Health Service (NHS) Foundation Trust and Newcastle University and the NIHR Biomedical Research Centre and Biomedical Research Unit in Dementia based at Cambridge University Hospitals NHS Foundation Trust and the University of Cambridge. (3) A study funded by Alzheimer’s Research UK, Alzheimer’s Society Doctoral Training Centre at Newcastle University, the Engineering and Physical Sciences Research Council of the UK, Wellcome Trust, Northumberland Tyne and Wear NHS Foundation Trust, NIHR Newcastle Biomedical Research Centre (BRC) based at Newcastle upon Tyne Hospitals NHS Foundation Trust and Newcastle University. These studies were approved by the corresponding NHS National Research Ethics committees.

### 2.2 Data acquisition

Structural MRI data were acquired using different scanners and protocols across three study cohorts. For the first cohort, MR imaging was performed on a 3T Siemens MAGNETOM Prisma Fit scanner using a T1-weighted three-dimensional magnetization-prepared rapid gradient-echo (3D-MPRAGE) sequence. Imaging parameters were as follows: repetition time (TR) = 2300 ms, echo time (TE) = 2.98 ms, inversion time (TI) = 900 ms, flip angle = 9°, GRAPPA acceleration factor = 2, voxel size = 1.0 × 1.0 × 1.0 mm³, field of view = 256 × 240 mm², and 160 sagittal slices. For the second and third cohorts, T1-weighted 3D-MPRAGE images were acquired on 3T Philips Achieva and Philips Intera Achieva systems. For the Philips Achieva scanner, imaging parameters included voxel size = 1.0 × 1.0 × 1.0 mm³, field of view ≈ 240 × 204 mm², and 180 slices (TR = 9.6 ms, TE = 4.6 ms, flip angle = 8°, SENSE acceleration factor = 2). For the Philips Intera Achieva scanner, similar parameters were used (TR = 8.3 ms, TE = 4.6 ms, flip angle = 8°, SENSE acceleration factor = 2), with voxel size = 1.0 × 1.0 × 1.0 mm³, field of view = 240 × 240 mm², and 180 slices. Note that TR values reported for Philips systems reflect sequence-specific definitions and are not directly comparable with Siemens MPRAGE TR values. After quality control, a total of 90 AD subjects and 105 LBD subjects were included. Demographic characteristics are summarized in Table 1. For statistical analyses of network metrics, only subjects with complete demographic information were included as covariates in the statistical models. Subjects with missing demographic data were excluded from group-level analyses but retained for classification analyses.

**Table 1.** Demographic characteristics of AD and LBD subjects.

Group	Total	Demographic	Age (mean ± SD)	Male	Female
AD	90	63	77.51±5.68	38	25
LBD	105	63	76.73±7.54	54	9

Age statistics are reported only for subjects with available demographic information.

### 2.3 Data preprocessing

Structural T1-weighted MR images were first visually inspected for major artifacts. All images were then resampled to an isotropic resolution of 1 mm using cubic interpolation to ensure consistent spatial resolution across subjects. To improve image quality, noise was reduced using a non-local means denoising algorithm adapted for Rician noise distributions (Coupé et al., 2008), which is well suited for MRI data. Intensity inhomogeneity caused by magnetic field non-uniformities was subsequently corrected using the N4 bias field correction algorithm (Tustison et al., 2010). Following these preprocessing steps, cortical surface reconstruction and anatomical parcellation were performed using the FreeSurfer software suite (version 8.1.0; Martinos Center for Biomedical Imaging, Boston, MA) (Fischl, 2012). The standard FreeSurfer pipeline includes skull stripping, intensity normalization, white matter segmentation, and reconstruction of the white and pial cortical surfaces, followed by topology correction to ensure a spherical surface representation (Dale et al., 1999). FreeSurfer also provides vertex-wise estimates of multiple cortical morphometric features (Fischl, Sereno, & Dale, 1999), including cortical thickness (CT), mean curvature (MC), gray matter volume (GV), surface area (SA), and sulcal depth (SD), which were used in subsequent analyses. To mitigate non-biological variability introduced by multi-site acquisition, scanner platform, and protocol differences, ComBat harmonization was applied to the vertex-wise morphometric features before network construction (Fortin et al., 2018). For each morphometric feature (m) and vertex (v), the observed value for subject (i) was modeled as

$$y_{imv}=\alpha_{mv}+{\text{x}}_{i}^{T}\beta_{mv}+\gamma_{b\left(i\right),mv}+\delta_{b\left(i\right),mv}\varepsilon_{imv}.$$ (1)

where *y_imv_* denotes the value of morphometric feature (m) at vertex (v) for subject (i), *α_mv_* is the overall mean, ${\text{x}}_{i}$ represents biological covariates of interest, and $\beta_{mv}$ denotes their corresponding effects. The term (b(i)) indicates the acquisition site batch for subject (i), while *γ_b_*_(_*_i_*_),_*_mv_* and *δ_b_*_(_*_i_*_),_*_mv_* represent additive and multiplicative batch effects, respectively. These batch effects were estimated using the empirical Bayes framework. The harmonized value was computed as

$$y_{imv}^{ComBat}=\left[y_{imv}-{\hat{\alpha }}_{mv}-{\text{x}}_{i}^{T}{\hat{\beta }}_{mv}-{\hat{\gamma }}_{b(i),mv}\right]/{\hat{\delta }}_{b(i),mv}+{\hat{\alpha }}_{mv}+{\text{x}}_{i}^{T}{\hat{\beta }}_{mv}.$$ (2)

Thus, site-related additive and multiplicative effects were removed while preserving biological variation associated with diagnosis and demographic covariates. The harmonized cortical thickness, mean curvature, gray matter volume, surface area, and sulcal depth features were subsequently used for landmark-based cortical similarity network construction. All key steps of the proposed framework, including 3HG extraction, thickness-constrained arealization, morphometric feature sampling, cortical similarity network construction, and graph theoretical analyses, were performed on each subject’s native cortical surface. Surface resampling to fsaverage7 was performed only as an additional step for group-level visualization of spatial maps. Quality control of cortical surface reconstruction was assessed using the Euler number, which provides an indicator of topological defects in the reconstructed cortical surfaces. Subjects were excluded if the Euler number of either hemisphere was lower than −100, indicating poor surface reconstruction quality.

### 2.4 3HG extraction

The three-hinge gyrus (3HG) is a cortical folding pattern defined by the convergence of three gyral ridges during cortical folding (H. Chen et al., 2017). This pattern exhibits strong consistency within species while showing substantial inter-individual variability (X. Li et al., 2017). Previous studies have demonstrated that 3HG regions possess distinctive structural and connectivity characteristics, including increased cortical thickness (K. Li et al., 2010), higher densities of diffusion MRI-derived fiber tracts (Ge et al., 2018), and greater diversity of structural and functional connectivity compared with other gyral regions (T. Zhang et al., 2020). These findings suggest that 3HGs may act as important connection hubs in cortical networks and play a key role in the organization of large-scale structural and functional brain systems.

**Fig. 2.**
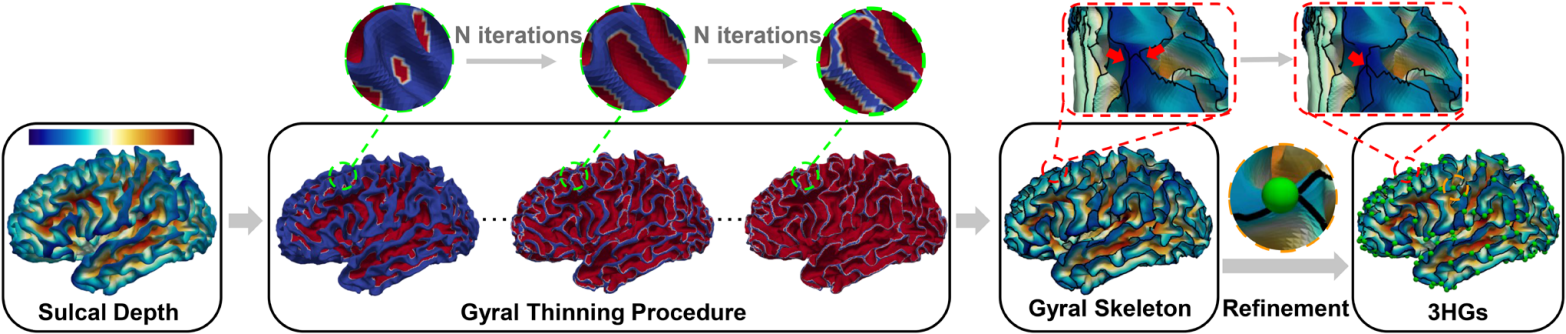
Pipeline for identifying 3HG landmarks from cortical surfaces. Sulcal depth and curvature are used to define gyral regions, which are iteratively thinned to obtain a gyral skeleton. The skeleton is refined to preserve meaningful ridges, and 3HGs are detected as junctions with three or more branches.

3HG landmarks were automatically extracted on the reconstructed white matter surface using a fully automated pipeline similar to that described in H. Chen et al. (2017). Cortical folding patterns were first characterized using vertex-wise SD and MC derived from FreeSurfer. Sulcal depth values were used to distinguish gyral and sulcal regions, with positive values indicating gyri and negative values indicating sulci. To improve robustness, isolated vertices were removed and minor inconsistencies in sulcal classification were corrected using local curvature information. A gyral thinning procedure was then applied to extract the central ridges of gyri. Specifically, gyral regions were iteratively thinned from sulcal boundaries under curvature constraints, progressively reducing each gyral region to a narrow ridge representing the gyral skeleton. This skeleton approximates the medial axis of gyral structures and captures the topological organization of cortical folding patterns. A skeleton graph was subsequently constructed by connecting neighboring vertices along the gyral skeleton. The graph was further refined by removing short branches and spurious connections to preserve only meaningful gyral ridges. In addition, long gyral ridges that were not initially connected to the skeleton were detected using curvature-based criteria and linked to the skeleton through shortest paths on the cortical surface. Finally, 3HGs were identified as vertices on the gyral skeleton with three or more incident branches, corresponding to junctions where multiple gyral ridges converge. To ensure topological consistency, nearby hinge pairs were further refined using surface-constrained shortest-path rewiring to eliminate redundant hinge detections. The resulting set of 3HG vertices represents individualized folding landmarks that capture key structural junctions within cortical folding patterns and were used as biologically meaningful nodes for subsequent network construction. An illustration of the 3HG identification pipeline is shown in Figure 2.

### 2.5 Cortical thickness-constrained arealization

To define localized cortical regions around each folding landmark, we performed a cortical thickness-constrained arealization procedure on the reconstructed cortical surface. Cortical thickness is closely related to underlying cytoarchitectonic organization and varies systematically across cortical areas, making it a useful proxy for identifying structurally coherent cortical territories (Demirci & Holland, 2022; Van Essen, 2020). By incorporating CT constraints during region growing, the resulting regions are encouraged to remain within morphometrically homogeneous cortical zones rather than expanding indiscriminately across folding boundaries (Petersen et al., 2024). Landmark vertices were treated as seeds, and regions were grown simultaneously from all seeds using a multi-source geodesic region-growing procedure. Geodesic distances were computed along the surface mesh based on Euclidean edge lengths. For each seed *s_i_*, the local thickness distribution was estimated from vertices within a small neighborhood surrounding the seed. Let *μ_i_* and *σ_i_* denote the mean and standard deviation of cortical thickness within this neighborhood. During region growing, a candidate vertex *v* was accepted into region *i* only if its thickness value satisfied a consistency constraint:

$$\left|t\left(v\right)-\mu_{i}\right|\leq \alpha \sigma_{i,\;}$$ (3)

where *t*(*v*) denotes the cortical thickness at vertex *v* and *α* is a tolerance parameter controlling the allowable deviation. The tolerance parameter *α* was selected based on a sensitivity analysis of arealized region-size stability and regional sampling adequacy. We tested α∈{0.1, 0.2, 0.3, 0.4, 0.5} across the study cohort. For each value, region-size stability was quantified using the coefficient of variation (CV) of arealized region sizes across landmarks within each subject, and sampling adequacy was quantified by the mean number of vertices per region. Smaller *α* values imposed stricter thickness constraints and produced smaller regions, which may reduce the reliability of per-region feature-distribution estimation. Larger *α* values increased region size but also tended to increase region-size heterogeneity. We selected α=0.3 because region size began to saturate around this value while the CV remained within a low range, providing a practical balance between sufficient vertices for regional feature estimation and stable region sizes (Supplementary Fig. S2). This value was, therefore, used in all subsequent analyses. This thickness-based gating prevents region propagation across areas with substantially different cortical thickness profiles. Region expansion proceeded iteratively along the surface mesh until a predefined spatial constraint was reached (5 mm in this study). When a vertex was reachable from multiple seeds and satisfied multiple gating criteria, it was assigned to the seed with the minimal geodesic distance:

$$s*\left(v\right)=\text{arg}\underset{i}{\min}D_{g}\left(v,s_{i}\right),$$ (4)

where *D_g_*(·) denotes geodesic distance on the cortical surface. To improve spatial coherence, two post-processing steps were applied. First, unlabeled vertex components fully enclosed by a single labeled region were filled to eliminate isolated holes. Second, a graph-based morphological closing operation was applied to smooth region boundaries and recover small gaps along the region periphery. Newly added vertices were assigned to the nearest labeled region through nearest-label propagation. The resulting vertex labels define a set of landmark-centered cortical regions that respect both surface geodesic constraints and local cortical thickness similarity, providing morphometrically coherent samples of cortical tissue for subsequent analysis.

### 2.6 Landmark-based cortical similarity network construction

Using the landmark-centered cortical regions obtained from the arealization procedure, we constructed a landmark-based cortical similarity network in which each landmark region represents a node and edges encode morphometric similarity between regions. For each vertex, a set of morphometric features (CT, GV, MC, SA, and SD) was used to characterize local cortical structure. To ensure comparability across features, each feature dimension was standardized across vertices using z-score normalization. For a given landmark region *R_i_*, the feature vectors of all vertices within the region form a multivariate sample:

$$X_{i}=\left\{x_{1},x_{2},\ldots ,x_{n_{i}}\right\},x_{k}\in {\mathbb{R}}^{d},$$ (5)

where *d* denotes the number of morphometric features. Following MIND networks (Sebenius et al., 2023), we quantified the divergence between regional feature distributions using a k-nearest-neighbor estimator of the Kullback–Leibler (KL) divergence. To improve estimation stability and avoid degenerate distances caused by repeated feature values, we fit a multivariate Gaussian kernel density estimate to each region’s feature distribution and drew *N* = 500 resampled points (Scott’s rule for bandwidth selection) before computing the divergence. Given two regions *R_i_* and *R_j_*, the KL divergence between their feature distributions is estimated as

$$D_{KL}\left(p_{i}∥p_{j}\right)=\frac{d}{n_{i}}\sum_{k=1}^{n_{i}}\text{log}\frac{s_{k}}{r_{k}}+\text{log}\frac{n_{j}}{n_{i}-1},$$ (6)

where *r_k_* denotes the Euclidean distance from sample *x_k_* to its second nearest neighbor within region *R_i_*, and *s_k_* denotes the distance from *x_k_* to its nearest neighbor in region *R_j_*. Because KL divergence is asymmetric, the symmetric form was used:

$$D_{KL}^{sym}\left(i,j\right)=D_{KL}\left(p_{i}∥p_{j}\right)+D_{KL}\left(p_{j}∥p_{i}\right).$$ (7)

Finally, morphometric similarity between regions was defined as

$$S_{ij}=\frac{1}{1+D_{KL}^{sym}\left(i,j\right)}$$ (8)

yielding a bounded similarity measure $S_{ij}\in \left(0,1\right]$. Applying this procedure to all landmark pairs resulted in a weighted similarity matrix *S*, representing the individualized landmark-based cortical similarity network. To ensure network connectivity while maintaining a fixed sparsity level across subjects, we used an MST-constrained proportional thresholding strategy. We first constructed a minimum spanning tree (MST) using the dissimilarity 1 − *S_ij_*, and then added the strongest remaining non-MST edges until the total number of edges reached 10% of all possible edges. Thus, the final graph density was fixed at 10%, including the MST edges.

### 2.7 Network metrics

To characterize the topological organization of the landmark-based cortical similarity networks, we computed a set of global and distribution-level graph metrics capturing complementary aspects of network integration, segregation, and hub organization.

#### 2.7.1 Global topology

Global network topology was quantified using standard graph theoretical measures. Network integration was assessed using global efficiency, defined as the average inverse shortest path length across all pairs of nodes:

$$E_{\text{glob}}=\frac{1}{N\left(N-1\right)}\sum_{i\neq j}\;\frac{1}{d_{ij}},$$ (9)

where *N* is the number of nodes and *d_ij_* denotes the shortest path length between nodes *i* and *j*. Characteristic path length was defined as the average shortest path length between all node pairs:

$$L=\frac{1}{N\left(N-1\right)}\sum_{i\neq j}d_{ij}.$$ (10)

Network segregation was assessed using both transitivity and the clustering coefficient. Transitivity was defined as the ratio of closed triplets to all connected triplets in the network. The clustering coefficient *C* was computed as the average local clustering coefficient across all nodes, $C=\frac{1}{N}\sum_{i=1}^{N}C_{i}$, where *C_i_* denotes the local clustering coefficient of node *i*. Degree assortativity was computed as the Pearson correlation coefficient between the degrees of connected node pairs, reflecting the tendency of nodes to connect with other nodes of similar degree. Small-world organization was quantified relative to matched random networks as

$$\sigma =\frac{C/C_{\text{rand}}}{L/L_{\text{rand}}},$$ (11)

where *C* and *L* denote the clustering coefficient and characteristic path length of the empirical network, respectively, and $C_{\text{rand}}$ and $L_{\text{rand}}$ denote the corresponding averages computed from degree-preserving random networks.

#### 2.7.2 Distribution-level metrics

To capture heterogeneity in node-level properties and hub organization, we further computed distribution-based metrics derived from nodal measures. Degree inequality was quantified using the Gini coefficient of the degree distribution,

$$G=\frac{\sum_{i=1}^{N}\sum_{j=1}^{N}\left|k_{i}-k_{j}\right|}{2N\sum_{i=1}^{N}k_{i}},$$ (12)

where *k_i_* denotes the degree of node *i*. Similarly, nodal efficiency inequality was computed using the Gini coefficient of nodal efficiency values. Hub dominance was assessed using the degree hubness ratio, defined as the ratio between the mean degree of the top 10% highest-degree nodes and the network-wide mean degree. Betweenness centrality dispersion was quantified as the 90th percentile of the betweenness centrality distribution, capturing the prominence of high-centrality nodes. Variability in nodal efficiency was further summarized by its standard deviation.

#### 2.7.3 Controlling for network size

An important methodological consideration in graph theoretical analysis of brain networks is that many topological metrics are strongly dependent on network size and density (Fornito et al., 2010). Previous work (Zalesky et al., 2010) has demonstrated that while qualitative properties of brain networks (e.g., the presence of small-world organization) are largely preserved across parcellation schemes, the quantitative values of graph metrics, such as clustering coefficient, characteristic path length, and global efficiency, can vary markedly as a function of the number of nodes. Consequently, differences in *N* across subjects or groups may lead to apparent differences in network topology that are purely driven by scale rather than underlying neurobiological effects. To mitigate this confounding effect, we explicitly controlled for network size in all statistical analyses. Specifically, *N* was included as a covariate in regression models (along with age and sex), ensuring that observed group differences in graph metrics reflect genuine alterations in network organization rather than trivial scaling effects. This control is particularly critical in the present study, where node definitions are derived from individualized cortical folding patterns, resulting in inherent variability in node count across subjects. By accounting for network size, our analysis enables a more interpretable and biologically meaningful comparison of topological properties across groups.

## 3 Results

### 3.1 Group differences in graph theoretical metrics

We first examined group differences in global graph theoretical metrics between AD and LBD using generalized linear models. The distributions of node count and average degree were largely overlapping between AD and LBD groups, with only a modest descriptive reduction in node count in AD relative to LBD (Supplementary Fig. S1). Average degree remained highly comparable between groups, consistent with the use of density-constrained thresholding.

**Fig. 3.**
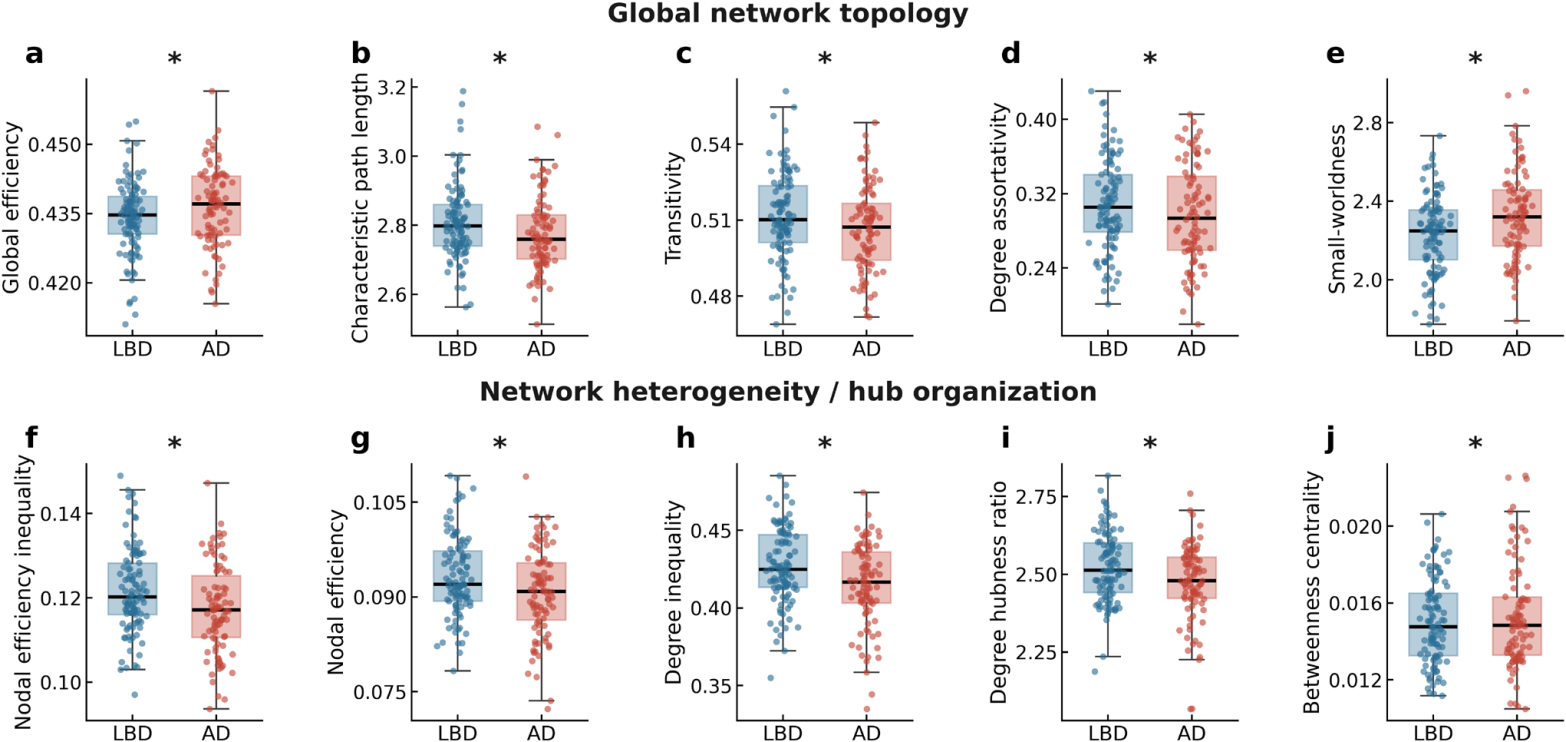
Group differences in global network topology, network heterogeneity, and hub organization metrics between AD and LBD. Boxplots show the distribution of graph theoretical metrics for LBD and AD groups, with individual subjects overlaid as points. Panels show (a) global efficiency, (b) characteristic path length, (c) transitivity, (d) degree assortativity, (e) small worldness, (f) nodal efficiency inequality, (g) nodal efficiency spread, (h) degree inequality, (i) degree hubness ratio, and (j) betweenness centrality. Asterisks indicate diagnosis effects surviving Benjamini–Hochberg FDR correction, controlling for age and sex (q<0.05 ).

We next assessed group differences in global network topology. As shown in Figure 3, several metrics exhibited significant differences between AD and LBD. LBD showed higher transitivity than AD (GLM p = 0.0248, FDR q = 0.0496) and longer characteristic path length (GLM p = 0.0034, FDR q = 0.0132). In contrast, AD showed higher small-worldness (GLM p = 0.0039, FDR q = 0.0132) and higher global efficiency (GLM p = 0.0040, FDR q = 0.0132). Together, these results indicate measurable group differences in both network segregation and integration, with LBD showing a tendency toward greater local clustering and reduced global integration, whereas AD showed relatively higher efficiency and small-world organization. We further examined hub organization and network heterogeneity metrics (Fig. 3f–j). Compared with AD, LBD exhibited higher degree inequality (GLM p = 0.0045, FDR q = 0.0223), higher degree hubness ratio (GLM p = 0.0145, FDR q = 0.0290), greater dispersion of nodal efficiency (GLM p = 0.0082, FDR q = 0.0249), a higher nodal efficiency inequality (GLM p = 0.0023, FDR q = 0.0223), and higher degree assortativity (GLM p = 0.0107, FDR q = 0.0267). Betweenness centrality also differed significantly between groups (GLM p = 0.01, FDR q = 0.0249), although the effect size appeared modest. Overall, these findings suggest that LBD networks exhibit a more heterogeneous and hub-dominated topological organization than AD networks.

**Fig. 4.**
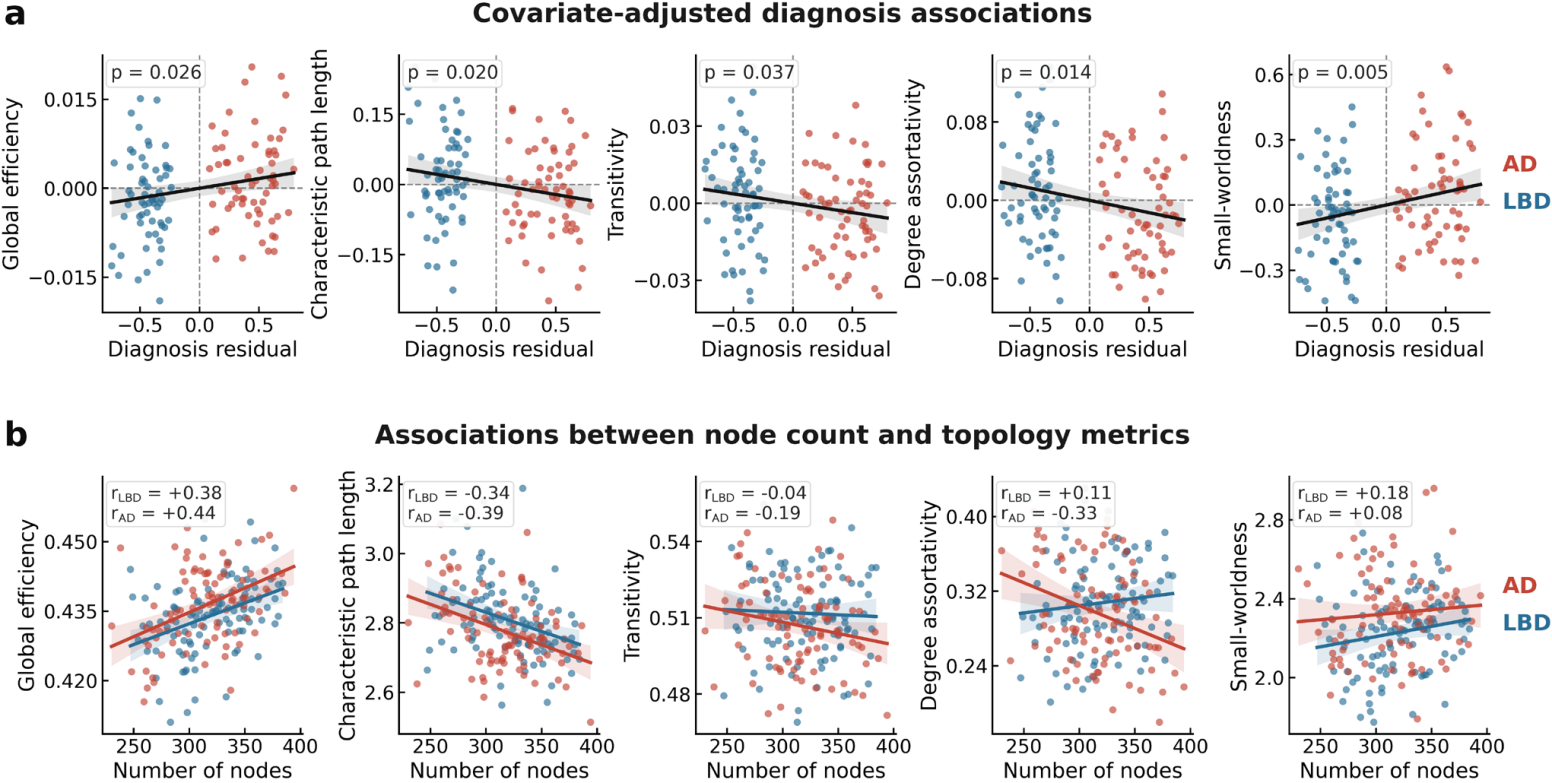
Covariate-adjusted diagnosis associations and node-count scaling effects for global network topology metrics. (a) Partial regression plots illustrating the association between diagnosis and global topology metrics after controlling for age, sex, and node count. P values indicate the diagnosis effect in the covariate-adjusted model. (b) Associations between the number of detected 3HG nodes and the corresponding global topology metrics. Correlation coefficients are reported separately for AD and LBD. Each point represents an individual subject, and solid lines denote fitted linear trends. AD is shown in red and LBD in blue.

Partial regression plots (Fig. 4a) further illustrated the covariate-adjusted associations between diagnosis and the major global metrics after controlling for age, sex, and node count. The residualized trends remained directionally consistent with the group-level comparisons, with AD associated with higher global efficiency and small-worldness and LBD associated with longer characteristic path length and higher transitivity.

To determine whether diagnosis effects were influenced by node-number variation, we repeated the group comparisons after including number of nodes as an additional covariate (Table 2). Several global metrics that were significant in the unadjusted models, including transitivity, characteristic path length, small-worldness, global efficiency, assortativity, and betweenness, no longer survived FDR correction after node adjustment. By contrast, nodal efficiency inequality, degree inequality, nodal efficiency, and degree hubness ratio remained significant, with only modest changes in effect size. Here, “attenuated by N adjustment” indicates that the diagnosis effect was significant before node-count adjustment but no longer survived FDR correction after including node count as a covariate. In contrast, “robust to N adjustment” indicates that the diagnosis effect remained significant after controlling for node count, suggesting that these metrics captured group differences beyond simple network-size scaling. These findings indicate that many apparent group differences in global topology are sensitive to node-count scaling, whereas several measures of topological heterogeneity remain relatively robust after controlling for network size.

**Table 2.** Effects of diagnosis (AD vs. LBD) on graph metrics before and after controlling for number of nodes.

	Unadjusted		Node-adjusted		
Metric	*β*	*q*	*β*	*q*	Interpretation
Transitivity	-0.008	0.050	-0.007	0.074	Attenuated by N adjustment
Characteristic path length	-0.056	0.013	-0.043	0.064	Attenuated by N adjustment
Small-worldness	0.121	0.013	0.120	0.053	Attenuated by N adjustment
Global efficiency	0.004	0.013	0.003	0.064	Attenuated by N adjustment
Assortativity	-0.026	0.027	-0.025	0.064	Attenuated by N adjustment
Nodal efficiency inequality	-0.006	0.022	-0.005	0.039	Robust to N adjustment
Degree inequality	-0.014	0.022	-0.014	0.039	Robust to N adjustment
Nodal efficiency	-0.003	0.025	-0.003	0.046	Robust to N adjustment
Degree hubness ratio	-0.059	0.029	-0.061	0.045	Robust to N adjustment
Betweenness	-0.001	0.025	-0.0003	0.352	Attenuated by N adjustment

Reported are standardized regression coefficients (*β*) and FDR-corrected *q* values from generalized linear models. The “Interpretation” column summarizes whether the diagnosis effect remained significant after including node count as an additional covariate. Metrics labeled “Robust to N adjustment” remained significant after FDR correction in the node-adjusted model (q<0.05), suggesting that the diagnosis effect was relatively independent of node-count scaling. Metrics labeled “Attenuated by N adjustment” no longer survived FDR correction after node adjustment, suggesting that the apparent diagnosis effect was partly explained by node-count-related scaling.

### 3.2 Node-count scaling effects on network topology

Because the proposed individualized networks are constructed from automatically detected folding landmarks, the number of nodes varies across subjects. We, therefore, examined whether graph metrics were associated with node count. Correlation analysis showed that several global topology measures were related to the number of detected nodes (Table 3). Across all subjects, global efficiency was positively correlated with node count (r = 0.395), whereas characteristic path length was negatively correlated (r = -0.352). By contrast, transitivity (r = -0.100), assortativity (r = -0.103), and small-worldness (r = 0.109) showed weaker overall associations. Similar patterns were observed within diagnostic subgroups, with the strongest and most consistent scaling effects seen for global efficiency (AD: r = 0.436, LBD: r = 0.381) and characteristic path length (AD: r = -0.391, LBD: r = -0.340). Assortativity showed a more heterogeneous relationship with node count across groups, being negative in AD (r = -0.330) but slightly positive in LBD (r = 0.108). These associations are illustrated in Figure 4b. Global efficiency increased with node count, whereas characteristic path length decreased as node count increased. Small-worldness showed a mild positive trend, while transitivity exhibited a weak negative trend. The association between assortativity and node count was less stable and appeared to differ by diagnostic group. Importantly, despite similar average degree across subjects due to density-constrained thresholding, substantial variation remained in the number of nodes, indicating that network size itself influenced multiple topological measures. Taken together, these results show that topology in individualized folding-based cortical similarity networks is partly shaped by node availability. This scaling effect is particularly relevant for interpreting group differences, because some apparent diagnostic effects may reflect variation in node count rather than disease-related topology alone.

**Table 3.** Correlation between network topology metrics and node count in the full cohort and diagnostic subgroups.

Network metric	All subjects	AD	LBD
Global efficiency	0.395	0.436	0.381
Characteristic path length	-0.352	-0.391	-0.340
Transitivity	-0.100	-0.192	-0.039
Assortativity	-0.103	-0.330	0.108
Small-worldness	0.109	0.081	0.176

### 3.3 Classification performance of topology and node-count features

**Fig. 5.**
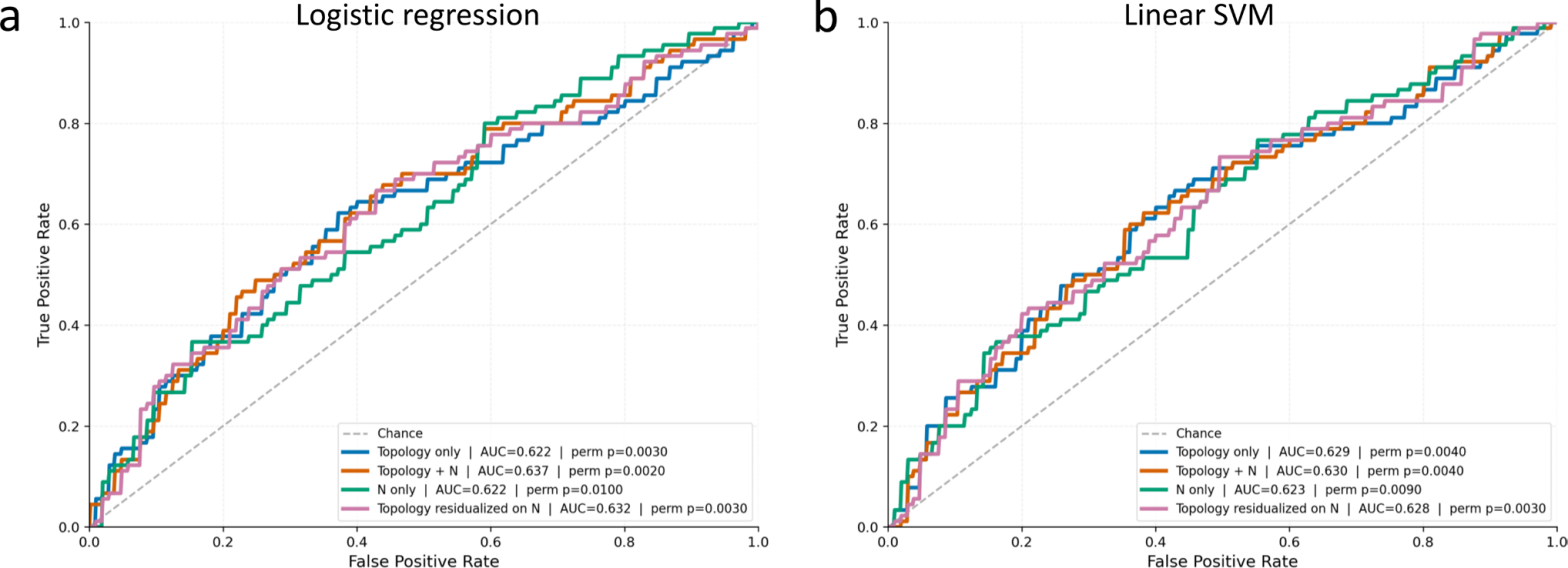
Receiver operating characteristic (ROC) curves for classification of AD vs. LBD. ROC curves obtained using nested cross-validation for (a) logistic regression and (b) linear SVM classifiers. Models were evaluated using different feature sets, including topology-only, topology + node count (N), N-only, and topology residualized with respect to N. Curves represent out-of-fold predictions, and the gray dashed diagonal line in each panel indicates the chance level.

We next evaluated whether topology-derived features could discriminate AD from LBD and whether node count contributed independent diagnostic information. Classification performance was assessed using nested cross-validation with an outer 10-fold loop and an inner 5-fold loop for model selection. Statistical significance was evaluated using permutation testing ($n_{\text{perm}}=1000$ ), and 95% confidence intervals for performance metrics were estimated using bootstrap resampling (*n* = 2000). Classification results are summarized in Table 4 and Figure 5. Using topology-only features, both L2-regularized logistic regression and linear SVM achieved above-chance discrimination between AD and LBD. Adding node count to the topological feature set yielded the highest overall performance, particularly for logistic regression, although the improvement over topology-only models was modest. Node-count-related features alone also achieved above-chance performance, indicating that node availability itself contains diagnostic information. Importantly, topological features remained informative even after residualizing out node-count effects. The residualized topology models achieved AUCs of 0.632 (95% CI [0.554,0.709], permutation p=0.0030 ) for logistic regression and 0.628 (95% CI [0.551,0.706], permutation p=0.0030 ) for SVM, remaining comparable with the topology-only models. Thus, although part of the discriminative signal is related to node-count variation, topological information beyond node count is still retained in the individualized folding-based networks. Overall, these results suggest that node count is not merely a nuisance variable but carries modest diagnostic information, while residualized topological patterns still provide weak but consistent discriminatory value for distinguishing AD from LBD. To assess whether the unbalanced sex distribution in the LBD group influenced the classification results, we performed an additional sensitivity analysis by residualizing topology-derived features with respect to sex. The sex-residualized topology features yielded comparable classification performance with the original topology features, with slightly higher AUC values for both logistic regression and linear SVM models (Supplementary Table S1). These results suggest that the observed AD–LBD classification performance was not solely driven by sex imbalance. A robustness analysis across nearby density thresholds showed that both the group-level graph-metric findings and the AD–LBD classification performance were stable across d=8%,10%,12% (Supplementary Fig. S3).

**Table 4.** Classification performance for AD vs. LBD under different feature-set definitions.

Feature	Model	AUC	Accuracy	Sensitivity	Specificity
Topology-only	LR	0.622 [0.542, 0.702]	0.617 [0.551, 0.685]	0.633 [0.533, 0.733]	0.600 [0.500, 0.698]
Topology-only	SVM	0.629 [0.553, 0.708]	0.614 [0.547, 0.684]	0.600 [0.494, 0.702]	0.629 [0.533, 0.725]
Topology + *N*	LR	0.637 [0.560, 0.714]	0.611 [0.543, 0.678]	0.622 [0.522, 0.725]	0.600 [0.505, 0.696]
Topology + *N*	SVM	0.630 [0.554, 0.708]	0.614 [0.546, 0.683]	0.600 [0.495, 0.701]	0.629 [0.535, 0.722]
*N*-only	LR	0.622 [0.548, 0.701]	0.568 [0.502, 0.642]	0.556 [0.455, 0.657]	0.581 [0.486, 0.679]
*N*-only	SVM	0.623 [0.547, 0.703]	0.557 [0.491, 0.632]	0.533 [0.432, 0.636]	0.581 [0.486, 0.674]
Topo res. *N*	LR	0.632 [0.554, 0.709]	0.602 [0.537, 0.672]	0.633 [0.536, 0.730]	0.571 [0.477, 0.670]
Topo res. *N*	SVM	0.628 [0.551, 0.706]	0.579 [0.513, 0.646]	0.578 [0.481, 0.678]	0.581 [0.489, 0.677]

Feature sets were defined as Topology-only, topological network metrics only; Topology + *N*, topological metrics plus node count; *N*-only, node-count-related features only; Topo res. *N*, topological metrics after residualizing out node count.

### 3.4 Spatial distribution of AD–LBD differences on the cortical surface

**Fig. 6.**
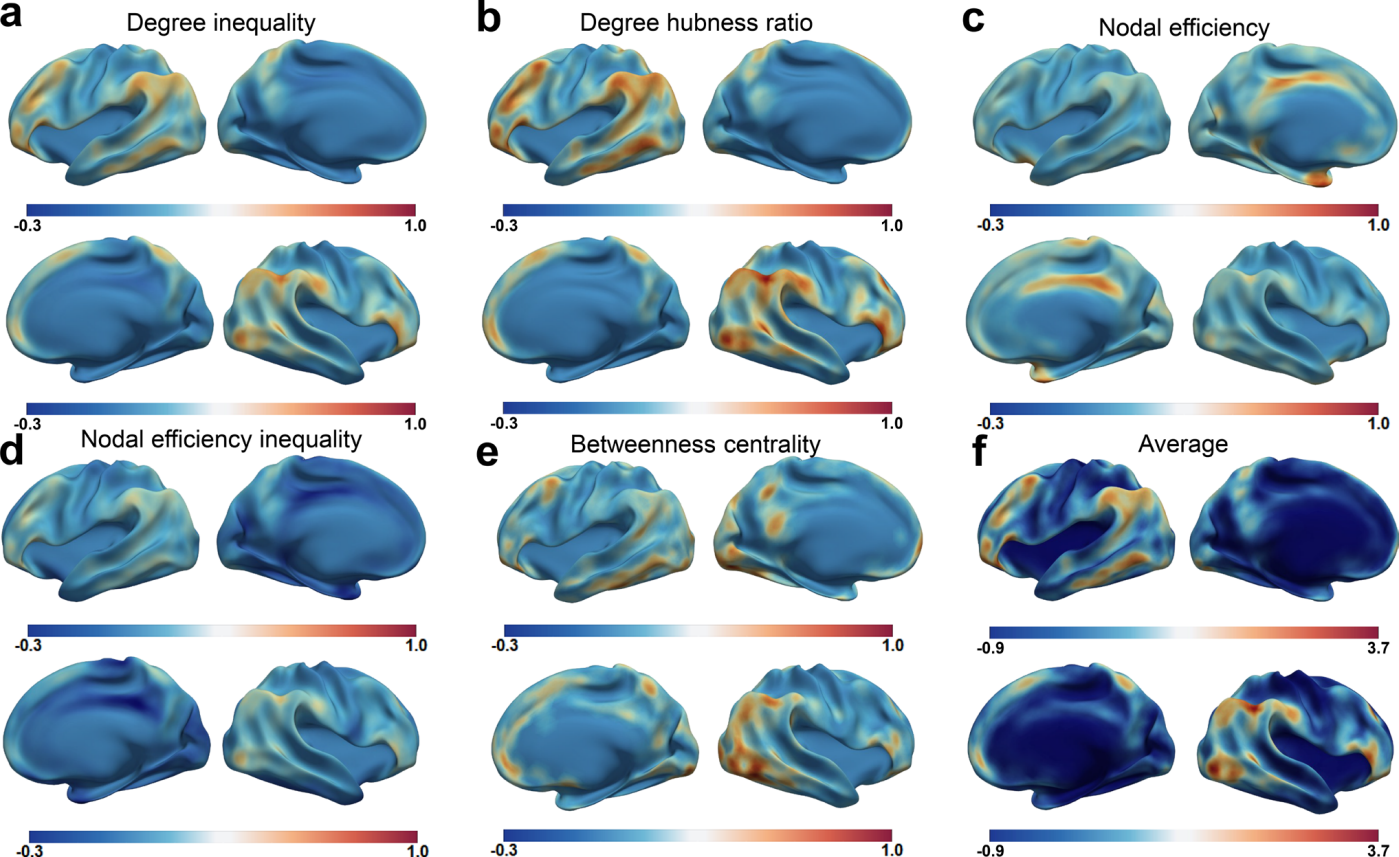
Spatial distribution of AD–LBD differences in node-level network metrics on the cortical surface. Surface projections of between-group difference magnitudes for network metrics derived from landmark-centered cortical areas in the folding-based cortical similarity network. Each mapped point corresponds to a thickness-constrained cortical area centered on a detected 3HG landmark and is displayed on inflated cortical surfaces in fsaverage space from multiple views. Maps are shown for (a) degree inequality, (b) degree hubness ratio, (c) nodal efficiency, (d) nodal efficiency inequality, (e) betweenness centrality, and (f) the average map. Color values indicate the relative magnitude of AD–LBD differences, with warmer colors representing cortical areas showing larger inter-group differences.

For group-level visualization, as shown in Figure 6, individualized landmark-level network measures were projected from native surface space to fsaverage7. Each subject’s 3HG-centered cortical regions were first defined in native space, and each region was assigned its corresponding area-level network value. The native-space regional values were then projected to fsaverage7 using FreeSurfer spherical registration (Fischl, Sereno, Tootell, & Dale, 1999). Because 3HG landmarks varied in number and location across subjects, we did not assume one-to-one landmark correspondence. Instead, projected values were aggregated on the common fsaverage7 surface within each diagnostic group, and AD–LBD spatial maps were computed as group-level differences in the aggregated surface values. Across metrics, the resulting maps showed spatially heterogeneous patterns rather than uniform differences across the cortical sheet. Degree inequality and degree hubness ratio exhibited relatively larger between-group differences over lateral temporal, parietal, and frontal cortical territories, with additional posterior cortical involvement. Nodal efficiency and nodal efficiency inequality showed broadly similar spatial distributions, with larger differences distributed across lateral temporo-parietal regions and extending into posterior cortex. Betweenness centrality also displayed a nonuniform anatomical pattern, with visually apparent contributions from lateral association cortex together with posterior occipital/parieto-occipital regions.

## 4 Discussion

In this study, we developed a fine-scale, individualized cortical similarity network framework based on automatically detected 3HG landmarks and used it to compare cortical network organization between AD and LBD. Three main findings emerged. First, multiple graph metrics were systematically associated with the number of detected folding landmarks, indicating that topology in individualized folding-based networks is partly shaped by node availability. Second, explicit adjustment for node count altered the interpretation of several diagnosis effects: many apparent group differences in global topology were attenuated after node adjustment, whereas several measures of topological heterogeneity remained significant. Third, despite these scaling effects, residualized topological patterns remained weakly but consistently informative for AD–LBD classification, and node count itself also carried modest diagnostic information. Together, these results suggest that individualized folding-based cortical similarity networks capture disease-related variation in cortical organization, but that this information must be interpreted in the context of node-count scaling.

### 4.1 Differential topological organization in AD and LBD

A central finding of this study is that AD and LBD differed not only in isolated morphometric properties, but also in the large-scale topological organization of individualized cortical similarity networks. Relative to AD, LBD showed longer characteristic path length and higher transitivity, together with higher degree inequality, degree hubness ratio, nodal efficiency dispersion, nodal efficiency inequality, betweenness centrality, and assortativity, whereas AD showed relatively higher global efficiency and small-worldness. Overall, these results suggest that, within the present folding-based network model, LBD is characterized by a more locally clustered but less globally integrated architecture, accompanied by greater topological heterogeneity and stronger concentration of network organization in a subset of highly connected or high-centrality regions. Importantly, the node-adjusted analyses showed that not all of these effects were equally robust. Several apparent group differences in global topology were attenuated after controlling for node count, whereas measures of topological heterogeneity such as degree inequality, degree hubness ratio, nodal efficiency, and nodal efficiency inequality remained significant. These results suggest that node-count scaling contributes substantially to the apparent differences in global topology, whereas heterogeneity-related effects distinguish AD from LBD more robustly.

Higher clustering or transitivity in this context should not be interpreted as beneficial. In diseased brain networks, increased clustering or transitivity can coexist with reduced integration, reflecting a less globally efficient and potentially more fragmented architecture rather than a healthier one (Bullmore & Sporns, 2009; Rubinov & Sporns, 2010). In the present study, the combination of higher transitivity, longer path length, and lower global efficiency in LBD is, therefore, more consistent with reduced large-scale communication efficiency than with preserved network optimality. Although our study examined structural cortical similarity networks rather than dynamic functional connectivity, the lower global efficiency observed in LBD is also broadly consistent with prior functional MRI findings suggesting reduced network flexibility in Lewy body disease (Schumacher et al., 2019). More broadly, the present topological differences are compatible with prior neuroimaging work showing relatively greater posterior cortical involvement in LBD and more prominent temporo-parietal and medial temporal pathology in AD (E. Burton et al., 2009; McKeith et al., 2017; Watson et al., 2012).

### 4.2 Hub heterogeneity as a distinguishing feature of LBD

One of the more distinctive aspects of the present results is that several metrics indexing heterogeneity and hub dominance were higher in LBD than in AD. Higher degree inequality and hubness ratio indicate a more uneven degree distribution, while increased nodal efficiency dispersion and inequality suggest that efficiency-related contributions are concentrated in a less homogeneous subset of nodes. Together, these findings point to a more heterogeneous allocation of topological roles in LBD. Notably, several of these effects remained significant after explicit adjustment for node count, suggesting that hub- and heterogeneity-related differences are not simply secondary consequences of network-size variation. This aspect of the results is particularly informative because it extends beyond the conventional integration–segregation axis. Much of the graph theoretical dementia literature has focused on metrics such as clustering, path length, and global efficiency, whereas the present findings suggest that the *distribution* of nodal importance may be at least as relevant as mean global topology. In other words, the difference between AD and LBD in this framework may not be captured solely by whether networks are globally more or less efficient, but also by how unevenly topological roles are allocated across the cortical sheet. This interpretation is conceptually consistent with the known clinical heterogeneity of LBD, which often combines fluctuating cognition, visuoperceptual dysfunction, and variable attentional impairments with a spatially heterogeneous pattern of cortical involvement (McKeith et al., 2017; Watson et al., 2012; Ye et al., 2020). Our findings, therefore, raise the possibility that topological heterogeneity may represent an imaging-level correlate of this broader clinical and anatomical heterogeneity, although testing such a link will require direct association with cognitive and clinical measures.

### 4.3 Node-count scaling as a key methodological issue in individualized networks

A major methodological finding of this study is that topology in individualized folding-based networks is partly shaped by node count. Because our networks were defined by subject-specific folding landmarks rather than a fixed atlas, the number of nodes varied across individuals. This variation was systematically associated with multiple graph metrics, most clearly global efficiency and characteristic path length, and more weakly with small-worldness, transitivity, and assortativity. These results are consistent with prior work showing that graph metrics depend strongly on network size, spatial scale, and density (Fornito et al., 2010; Van Wijk et al., 2010; Zalesky et al., 2010). In particular, Zalesky et al. (2010) demonstrated that although broad organizational features such as small-world structure may be preserved across scales, the numerical values of efficiency, clustering, path length, and small-worldness can vary substantially with node definition. Our findings extend this issue from atlas-based parcellations to individualized folding-derived networks. Here, node-count variation is not simply a consequence of analyst-defined parcellation resolution, but arises from subject-specific cortical geometry and potentially from disease-related changes that affect landmark availability (M. Chen, Chen, Cao, Zhang, Su, et al., 2026). This makes node count both a confound and a biologically meaningful variable. If ignored, node-count differences can materially alter the interpretation of diagnosis effects. In the present study, several group differences in global topology no longer survived FDR correction after node adjustment, whereas multiple heterogeneity-related metrics remained significant with only limited changes in effect size. Our results, therefore, support treating node count as an explicit part of the model rather than as a purely technical nuisance. More broadly, this highlights a central trade-off in individualized connectomics. Fixed atlases facilitate between-subject comparison but may obscure subject-specific cortical organization (Fischl et al., 2008). Individualized landmark-based networks preserve such anatomical specificity, but they also introduce scale variability that must be accounted for statistically. Future studies using individualized morphometric or folding-based networks should, therefore, report node-count distributions, quantify node-count–metric relationships, and test whether observed group effects remain after adjusting for network size.

### 4.4 Residualized topology and node count carry complementary diagnostic information

The classification analyses provide an informative complement to the univariate statistics. Topology-only models achieved above-chance discrimination between AD and LBD, but performance was modest. Adding node count yielded the highest AUC, while node-count-only models also performed above chance. Notably, topological features retained discriminatory value even after residualizing out node-count effects. These findings support two conclusions. First, node count itself contains disease-related information. This is consistent with the idea that the availability of individualized 3HG landmarks is not random, but is partly shaped by the structural state of the cortex. Because gyral landmarks arise from folding geometry, their detectability and local arealization may be altered by disease-related morphometric change, regional thinning, or greater anatomical simplification. While the present study cannot identify the exact biological determinants of reduced or altered landmark availability, the fact that node count alone performed above chance suggests that this quantity captures more than a purely procedural characteristic of graph construction. Second, residualized topology remained informative, indicating that AD–LBD differences were not reducible to node-count scaling alone. This is important because the goal of the present study was not primarily to maximize classification performance, but to evaluate whether the proposed folding-based network representation retains disease-relevant organizational information after accounting for network-size effects. The persistence of above-chance classification after residualization suggests that these individualized networks encode aspects of cortical organization that are informative for disease differentiation beyond simple variation in number of nodes. In this sense, the classification results serve mainly as evidence that the proposed representation preserves nonredundant disease-related information, complementing our prior work in which individualized folding-based networks were used more directly for predictive modeling (M. Chen, Chen, Cao, Zhang, Liu, et al., 2026; T. Chen, Chen, Zhuang, et al., 2025).

### 4.5 Spatially structured differences implicate temporal, temporoparietal, and posterior cortical systems

The surface projections provide an anatomical complement to the graph-level findings by indicating where AD–LBD differences in area-level network measures are most strongly expressed. Rather than being uniformly distributed across the cortical sheet, these differences showed a clear spatial structure, with prominent effects in lateral temporal, temporo-parietal, and posterior cortical territories, including occipital and parieto-occipital regions. This distribution is unlikely to reflect a nonspecific consequence of the mapping procedure alone, as the strongest effects were concentrated in cortical systems that have repeatedly been implicated in the differential neuroimaging profiles of AD and LBD. The temporal lobe effects are of particular interest. The high-value regions in middle and inferior temporal cortex suggest that AD–LBD differences in network organization are prominently expressed in lateral temporal association areas. This interpretation is broadly consistent with previous structural imaging studies indicating that temporal lobe involvement differs between the two disorders, even though the exact anatomical emphasis may vary across cohorts (Barber et al., 2001; E. Burton et al., 2009; Vemuri et al., 2011; Yousaf et al., 2019). Although the present surface projections do not directly measure regional atrophy, they suggest that disease-related differences in folding-based network organization are partly anchored in temporal cortical systems known to be affected in both AD and LBD. The posterior cortical effects are likewise biologically plausible. High-value regions in occipital and parieto-occipital cortex are consistent with the well-established posterior vulnerability of LBD (Harper et al., 2017; Yousaf et al., 2019). Structural MRI studies have reported posterior cortical and occipital involvement in LBD, and PET studies have repeatedly identified occipital hypometabolism as a characteristic feature of Lewy body disease (Hamed et al., 2018; Kantarci et al., 2012; Watson et al., 2012). The correspondence between our network-difference maps and these posterior imaging signatures suggests that the proposed folding-based representation is sensitive to disease-related alterations in cortical systems that are especially relevant to LBD. At the same time, the temporo-parietal high-value regions are also compatible with the established anatomical profile of AD. Structural MRI studies consistently show that AD is associated with widespread cortical loss involving temporal and parietal association cortices, in keeping with the known distribution of AD-related neurodegeneration (Blanc et al., 2015; Jacobs et al., 2012; Watson et al., 2015). The present maps, therefore, likely reflect the superimposition of two partially overlapping but distinct disease-related patterns: greater temporo-parietal involvement associated with AD and greater posterior cortical involvement associated with LBD. From this perspective, the value of the surface projections lies not in identifying a single disease-specific hotspot, but in showing that the topological differences captured by the folding-based network are embedded in anatomically meaningful large-scale cortical systems. Interestingly, this spatial pattern partially converges with recent developmental MSN findings. Yan et al. (2026) reported that cognition-associated MSN patterns in adolescents were mainly located in frontal, temporal, and occipital cortices and showed age-dependent changes. This overlap suggests that the regions highlighted in our AD–LBD maps may belong to cognition-related association systems that are sensitive to structural change across the lifespan, undergoing refinement during development while remaining vulnerable to aging- and disease-related disruption. This convergence should, therefore, be interpreted cautiously as overlap at the level of large-scale cortical systems, rather than evidence for identical mechanisms across development and dementia. More generally, these spatial patterns should be interpreted as descriptive projections of between-group difference magnitude rather than as vertex-wise inferential statistics. They are, therefore, most informative as anatomically grounded visualizations of where disease-related network differences are concentrated, rather than as a precise localization of cortical pathology.

### 4.6 Why a folding-based individualized MSN framework may be useful

The present study also has implications beyond dementia. A major challenge for structural connectomics is how to define nodes in a way that is both anatomically meaningful and comparable across individuals. Traditional atlas-based approaches enforce common dimensionality but may obscure subject-specific folding geometry and fine-scale structural variation. Morphometric similarity methods have already shown that inter-regional similarity in MRI-derived features tracks cytoarchitectonic organization, gene co-expression, and axonal connectivity (Sebenius et al., 2023, 2025; Seidlitz et al., 2018). Our framework builds on that logic by redefining nodes around individualized folding landmarks. This is attractive conceptually because cortical folding is not merely a nuisance for alignment, it is itself related to cortical architecture and connectivity (Fischl et al., 2008; Van Essen, 1997, 2020). The use of thickness-constrained arealization is an additional strength. By restricting region growth according to local cortical thickness similarity, the method attempts to define local samples that are more morphometrically coherent than simple radius-based neighborhoods. This is particularly relevant given evidence that cortical thickness varies systematically with local curvature and depth (Demirci & Holland, 2022; S. Wang et al., 2021). Although this approach does not solve the node-definition problem entirely, it offers a biologically motivated alternative to conventional atlas parcels and may be especially useful in settings where disease processes alter local cortical geometry in individualized ways.

### 4.7 Limitations

Several limitations should be noted. First, the present individualized network representation focuses specifically on gyral folding landmarks, namely 3HG-centered cortical areas. While this provides a biologically motivated and individualized basis for network construction, it captures only part of cortical folding architecture. Future work could extend this framework to other cortical landmarks, such as gyral peaks (S. Zhang et al., 2022, 2023) or sulcal pits (Auzias et al., 2015; Im & Grant, 2019), or to more spatially comprehensive whole-cortex folding representations that jointly model gyral and sulcal organization (Cao et al., 2026). Second, the current analysis was limited primarily to global topology and single-node level effects. As a result, intermediate mesoscale organization remains unexplored. Future studies could build on recent 3HG community modeling work (M. Chen, Chen, Zhuang, et al., 2026; M. Chen et al., 2025; Zhuang et al., 2025) to investigate whether disease-related effects are preferentially expressed at the level of folding-based subnetworks. Third, the present study was cross-sectional and, therefore, cannot determine whether the observed topological differences reflect stable disease traits, stage-dependent changes, or progressive network reorganization over time. Longitudinal analyses will be important for clarifying how folding-based network organization evolves across disease progression. Although ComBat harmonization was applied to reduce non-biological variability related to multi-site acquisition, scanner platform, and protocol differences, residual site effects may still remain, particularly because acquisition protocols and diagnostic group composition were not fully balanced across cohorts. Future studies using larger and prospectively harmonized multi-site datasets are needed to further validate the robustness and generalizability of these findings. And also, the LBD group showed an unbalanced sex distribution, with substantially fewer female participants than male participants. Although sex was included as a covariate in the statistical models and the sex-residualized sensitivity analysis yielded comparable classification performance, the limited number of female LBD participants restricts our ability to evaluate sex-specific network alterations. Future studies with larger and more sex-balanced cohorts are needed to further validate these findings.

### 4.8 Conclusions

In summary, this study shows that individualized gyral folding-based cortical similarity networks provide a biologically grounded framework for investigating cortical organization in AD and LBD. The results indicate that AD and LBD differ in both global topology and hub-related heterogeneity, with LBD showing a more uneven and less globally integrated network configuration within the present framework. At the same time, topology in these individualized networks is partly constrained by node-count scaling, making explicit control of network size essential for valid group comparison. Residualized topology and node count each carried modest but nonredundant diagnostic information, and their cortical projections revealed anatomically structured between-group differences across distributed association and posterior cortex. More broadly, these findings argue that individualized, folding-informed network models can reveal disease-related structural organization that is not easily captured by fixed atlas-based representations, provided that their inherent scaling properties are treated as a central part of the analysis rather than as an afterthought.

## Supplementary Material

Supplementary Material

## Data Availability

The data used in this study are not publicly available due to ethical and privacy restrictions but are available from the corresponding author upon reasonable request and with permission from the data providers. The code used to generate the results in this study is publicly available at https://github.com/m1nhengChen/Gyral-Folding-Network.
