# Supplementary material for: Fine-scale individualized gyral folding-based cortical similarity networks reveal distinct organizational patterns in Alzheimer’s disease and Lewy body dementia
Source: Imaging Neurosci (Camb). 2026 Aug 6;4:IMAG.a.1322. doi: 10.1162/IMAG.a.1322 (PMC13449931; doi:10.1162/IMAG.a.1322)
Supplement: Supplementary file 1 [file IMAG.a.1322_supp.pdf]

Supplementary Material for
Fine-scale individualized gyral folding-based cortical similarity networks reveal distinct organizational patterns in Alzheimer's disease and Lewy body dementia

Minheng Chen,[1] Chao Cao,[1] Tong Chen,[1] Dunia Alhamad,[2,3] Tianming Liu,[4] Li Su,[2,5] and Dajiang Zhu[1*]

[1]Department of Computer Science and Engineering,
University of Texas at Arlington, Arlington, TX 76019, USA
[2]Sheffield Institute for Translational Neuroscience,
University of Sheffield, Sheffield, UK
[3]King Saud Bin Abdulaziz University for Health Sciences (KSAU-HS),
Ministry of National Guard Health Affairs, Riyadh, Kingdom of Saudi Arabia
[4]School of Computing, University of Georgia, Athens, GA 30602, USA
[5]Department of Psychiatry, University of Cambridge, Cambridge, UK

[*]Correspondence: dajiang.zhu@uta.edu

S1 Supplementary Tables

Table S1: Sensitivity analysis assessing the influence of sex imbalance on AD vs. LBD classification.

**Feature set**	**Model**	**Adj.**	**AUC**	**Accuracy**	**Sensitivity**	**Specificity**	p_{perm}
Topology	LR	None	0.624 [0.523, 0.719]	0.619 [0.533, 0.699]	0.619 [0.500, 0.734]	0.619 [0.492, 0.732]	0.030
Topology	SVM	None	0.600 [0.500, 0.698]	0.595 [0.513, 0.677]	0.587 [0.469, 0.710]	0.603 [0.482, 0.721]	0.050
Topology res. sex	LR	Sex	0.647 [0.549, 0.740]	0.651 [0.571, 0.730]	0.651 [0.536, 0.766]	0.651 [0.529, 0.762]	0.011
Topology res. sex	SVM	Sex	0.645 [0.549, 0.741]	0.643 [0.562, 0.725]	0.635 [0.517, 0.750]	0.651 [0.529, 0.763]	0.019

Feature sets were defined as follows: Topology + local, topology-derived global and local network metrics without additional sex residualization; Topology + local res. sex, the same feature set after residualizing out sex. Values in brackets indicate 95% confidence intervals estimated using 2,000 bootstrap resamples. Permutation-based p-values were estimated using 1,000 label permutations. All models were evaluated using nested cross-validation with an outer 10-fold loop and an inner 5-fold loop. The analysis included 127 subjects, with 63 AD and 64 LBD subjects.

S2 Supplementary Figures

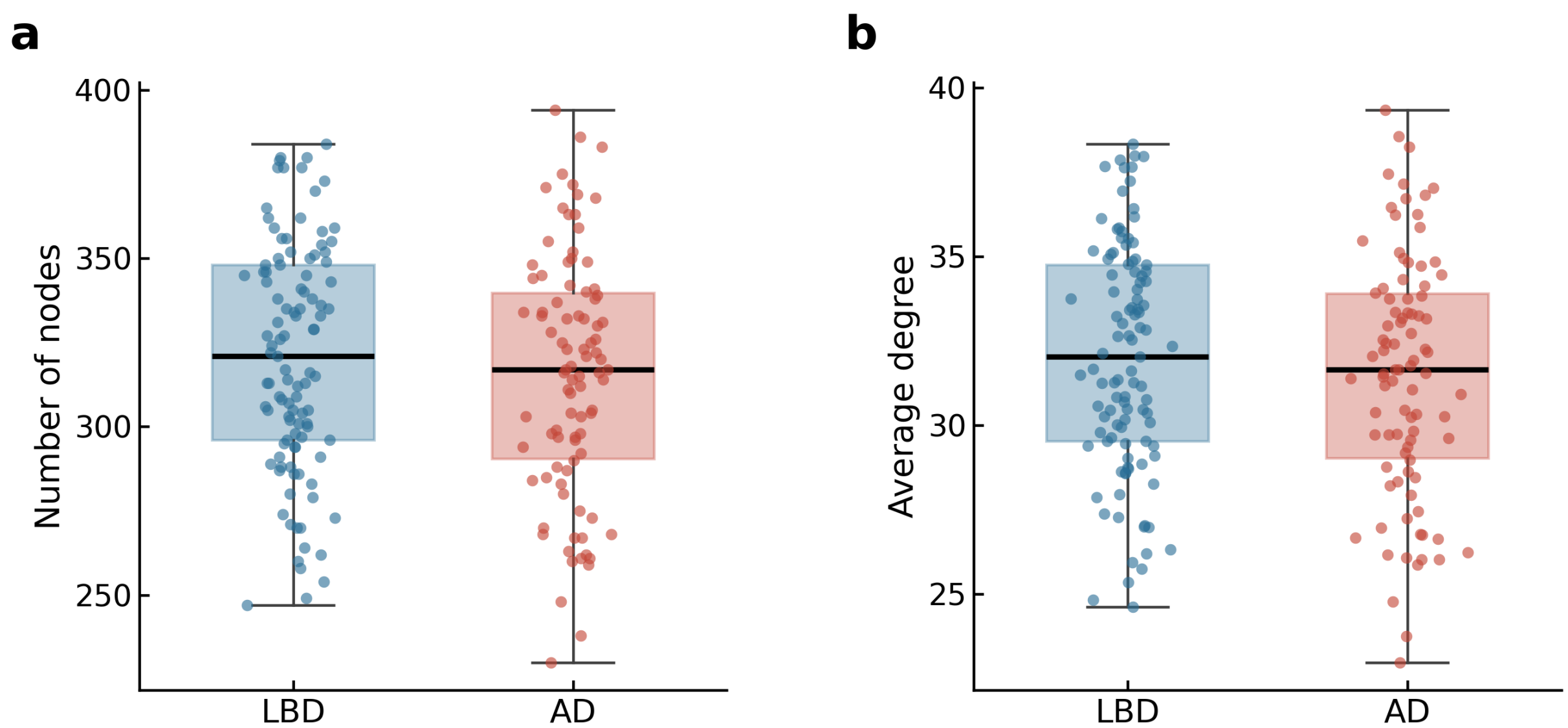

Figure S1: Node count and average degree across groups. **a** distribution of the number of nodes (N) in cortical similarity networks for AD and LBD. **b** distribution of average node degree. While the overall distributions are largely overlapping between groups, AD shows a modest reduction in node count compared to LBD. Average degree remains comparable due to density-constrained thresholding. Boxplots indicate median and interquartile range, with overlaid points representing individual subjects.

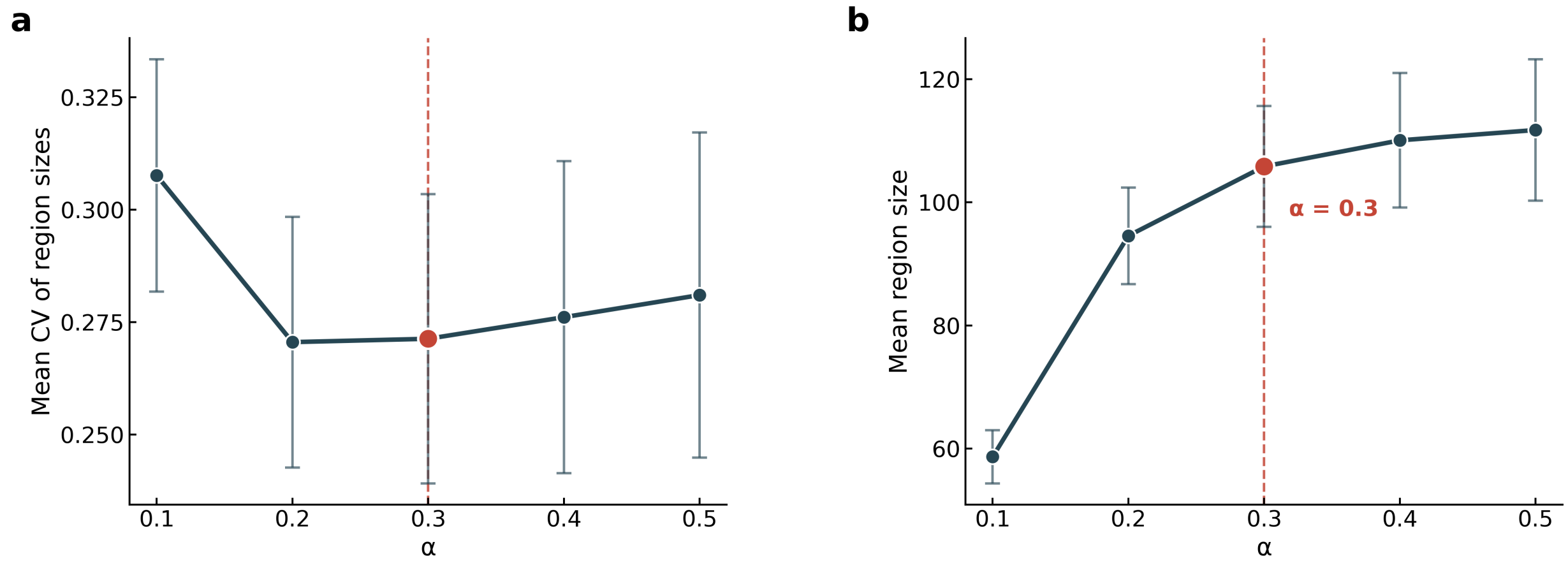

Figure S2: Sensitivity analysis for the tolerance parameter α in thickness-constrained arealization. **a** Mean coefficient of variation (CV) of arealized region sizes across subjects. **b** Mean number of vertices per region across α. Error bars indicate variability across subjects. Very small α values produced relatively small regions, whereas larger values increased the number of vertices per region but also increased region-size variability. The selected value $\alpha = 0.3$ lies near the region-size saturation knee while maintaining relatively low region-size CV, providing a practical balance between regional sampling adequacy and region-size stability.

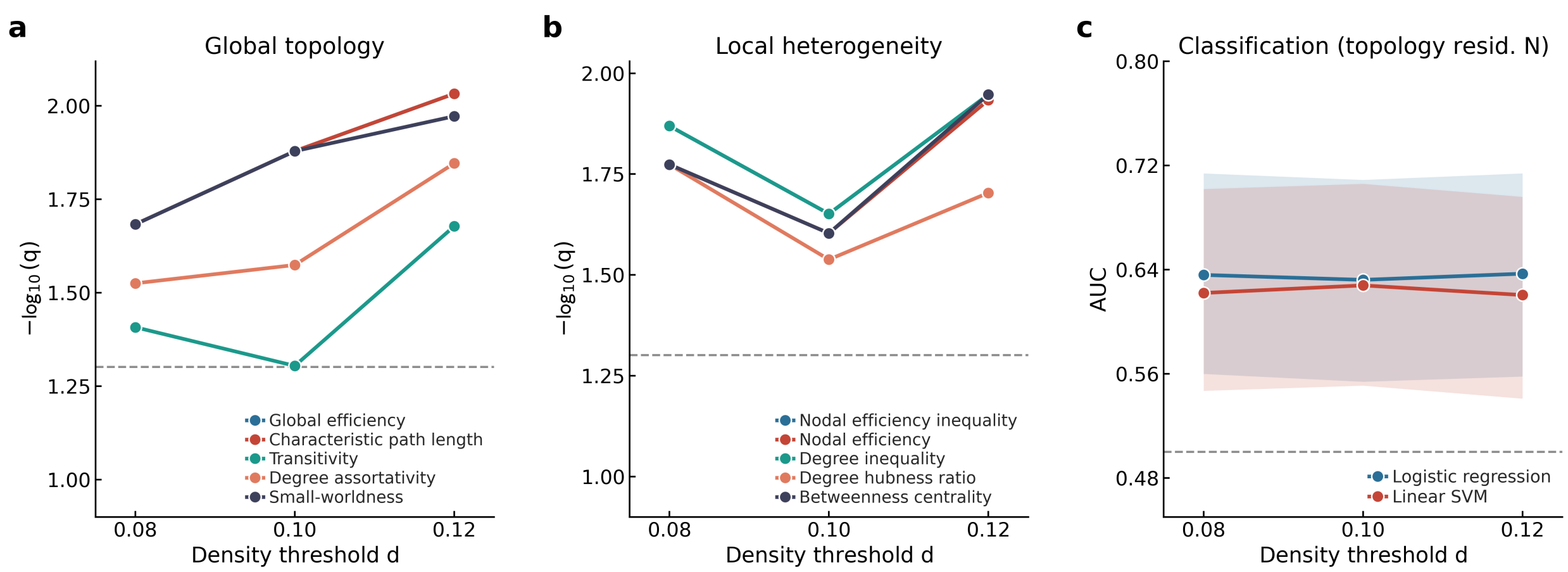

Figure S3: Robustness of the main results across nearby density thresholds ($d = 8\%, 10\%, 12\%$). **a**-**b** $-\log_{10}(q)$ values for the diagnosis term in generalized linear models ($\text{metric} \sim \text{dx} + \text{age} + \text{sex}$) for **a** five global topology metrics and **b** five local heterogeneity/hub metrics. The dashed line indicates the significance threshold corresponding to $q = 0.05$ after Benjamini-Hochberg FDR correction. All ten metrics remained significant across the three density thresholds. **c** Nested cross-validation AUC for AD vs. LBD classification using the topology-residualized-on-N feature set, shown with 95% bootstrap confidence intervals (shaded). Both classifiers (logistic regression and linear SVM) showed nearly identical performance across the three density thresholds (AUC variation < 0.005), indicating that the discriminative signal was not driven by the specific edge-density choice.
